# Tensin-3 is involved in osteogenic versus adipogenic fate of human bone marrow stromal cells

**DOI:** 10.1007/s00018-023-04930-5

**Published:** 2023-09-05

**Authors:** Shuang Zhang, Jeroen van de Peppel, Marijke Koedam, Johannes P. T. M. van Leeuwen, Bram C. J. van der Eerden

**Affiliations:** Laboratory for Calcium and Bone Metabolism, Department of Internal Medicine, Erasmus MC, Erasmus University Medical Center, Doctor Molewaterplein 40, 3015GD Rotterdam, The Netherlands

**Keywords:** TNS3, Bone marrow-derived mesenchymal stromal cells, Osteogenic differentiation, Adipogenic differentiation, RhoA activity, Cytoskeleton

## Abstract

**Background:**

The tightly controlled balance between osteogenic and adipogenic differentiation of human bone marrow-derived stromal cells (BMSCs) is critical to maintain bone homeostasis. Age-related osteoporosis is characterized by low bone mass with excessive infiltration of adipose tissue in the bone marrow compartment. The shift of BMSC differentiation from osteoblasts to adipocytes could result in bone loss and adiposity.

**Methods:**

*TNS3* gene expression during osteogenic and adipogenic differentiation of BMSCs was evaluated by qPCR and Western blot analyses. Lentiviral-mediated knockdown or overexpression of *TNS3* was used to assess its function. The organization of cytoskeleton was examined by immunofluorescent staining at multiple time points. The role of TNS3 and its domain function in osteogenic differentiation were evaluated by ALP activity, calcium assay, and Alizarin Red S staining. The expression of Rho-GTP was determined using the RhoA pull-down activation assay.

**Results:**

Loss of TNS3 impaired osteogenic differentiation of BMSCs but promoted adipogenic differentiation. Conversely, TNS3 overexpression hampered adipogenesis while enhancing osteogenesis. The expression level of TNS3 determined cell shape and cytoskeletal reorganization during osteogenic differentiation. TNS3 truncation experiments revealed that for optimal osteogenesis to occur, all domains proved essential. Pull-down and immunocytochemical experiments suggested that TNS3 mediates osteogenic differentiation through RhoA.

**Conclusions:**

Here, we identify TNS3 to be involved in BMSC fate decision. Our study links the domain structure in TNS3 to RhoA activity via actin dynamics and implicates an important role for TNS3 in regulating osteogenesis and adipogenesis from BMSCs. Furthermore, it supports the critical involvement of cytoskeletal reorganization in BMSC differentiation.

**Supplementary Information:**

The online version contains supplementary material available at 10.1007/s00018-023-04930-5.

## Background

Osteoporosis is a skeletal disorder characterized by reduced density of mineralized bone, leading to decreased mechanical strength and increased fracture risk [1]. This reduced mineral density is caused by remodeling abnormalities associated with uncoupling between bone formation and resorption [2]. Bone marrow mesenchymal stromal cells (BMSCs) are multiple lineage progenitors capable of differentiating into osteoblasts and adipocytes [3, 4]. Emerging evidence implicates that the spatio-temporal control of the balance between osteogenesis and adipogenesis contributes to skeletal health [5, 6]. The cellular fate decision process towards these lineages is closely linked and considered to be inversely regulated, as osteogenic differentiation of MSCs is typically accompanied by inhibition of adipogenesis [7, 8]. The allocation of MSCs to either osteoblasts or adipocytes is strictly orchestrated by several molecular factors. Transcription factors such as runt-related transcription factor 2 (RUNX2) and osterix (SP7) are considered master regulators to govern osteogenesis [9, 10], while CCAAT/enhancer binding proteins (C/EBPs) and peroxisome proliferator-activated receptor gamma (PPAR-γ) determine adipogenic differentiation [11, 12]. Other lineage-specific factors, such as collagen type 1 (COL1A1) and osteocalcin (BGLAP) participate in mature differentiation of osteoblasts and Acyl-CoA-binding protein (ACBP) and fatty acid synthase (FASN) are involved in adipocyte differentiation [12–15]. The identification of these molecular switches is vital to develop therapeutic strategies to overcome aberrant lineage commitment in osteoporosis and other relevant bone-related diseases.

Tensins (TNS) are a family of focal adhesion proteins consisting of four members, namely tensin-1, -2, -3 and C-terminal tensin-like (CTEN) [16]. All four tensins harbor an Src homology (SH) 2 and a phosphotyrosine-binding (PTB) domain, which are the unique structural features of the tensin family [16]. CTEN, in contrast to the other tensins, lacks the actin-binding domain (ABD) located at the N-terminus, making it the shortest member within the tensin family [16]. These different domains allow tensins to bridge the extracellular matrix with the cytoskeletal networks through integrin receptors and other protein complexes, and to play essential roles in signal transduction pathways and cytoskeletal reorganization [16, 17]. In recent years, studies involving tensins, especially tensin-3 (TNS3), mainly focused on tumorigenesis and metastasis, showing that its dysregulation is closely associated with multiple cancers including lung cancer, breast cancer and kidney cancer [18–20]. Interestingly, inactivation of *Tns3* results in growth retardation and postnatal lethality in homozygous mutant mice with fewer proliferating cells present in the resting zone of the epiphyseal growth plate, indicating incomplete development of the skeleton [21]. Moreover, Tns3 cooperates with Dock5 to regulate podosome, a specialized adhesion structure involved in osteoclast activity, reorganization in RAW264.7 cells [22]. Human genome-wide association study identified *TNS3* (lead variant at rs6949739) is associated with height, [23] further suggesting the involvement of TNS3 in the skeleton and supporting the growth plate observations in knockout mice [25]. In view of the potential role of TNS3 in skeletal development, and the importance of the lineage commitment process during bone formation, studies regarding the function and regulation of TNS3 in BMSCs differentiation are needed.

In the present study, we identified TNS3 is involved in osteogenic and adipogenic differentiation in BMSCs using *TNS3* loss- and gain-of-function models, demonstrating its important role in the determination of lineage commitment. Our work implies that TNS3 is a potential target for the development of bone anabolic strategies.

## Methods

### Cell culture and differentiation

Human bone marrow-derived mesenchymal stromal cells from a 33-year-old male (BMSCs, Lonza, Basel, Switzerland), tested for tri-lineage differentiation into osteoblasts, adipocytes, and chondrocytes, and being positive for CD105, CD166, CD29, and CD44, and negative for CD14, CD34, and CD45, were cultured as described previously [24–26]. Two days after seeding, BMSCs were initiated to osteogenic differentiation using 100 nM dexamethasone and 10 mM β-glycerophosphate. For adipogenic differentiation, cells were induced with adipogenic induction medium consisting of 100 nM dexamethasone, 60 μM indomethacin, and 0.5 mM 3-isobutyl-1-methylxanthine. Cells used in the experiments were at passage 7, and media were refreshed every 3 or 4 days.

### Generation of constructs and lentivirus-mediated knockdown and overexpression

Short hairpin RNAs (shRNAs) targeting *TNS3* were purchased from TRC-1.0 library (Sigma-Aldrich, Zwijndrecht, The Netherlands; Table S1). Non-targeting shRNA with a scrambled sequence was used as a control. To achieve *TNS3* overexpression, full-length human *TNS3* cDNA (Horizon Discovery, Waterbeach, UK) was cloned into a pEntr vector and further transferred to a pLenti6.3/V5–DEST vector (Gateway Vector Kits, Life Technologies Europe B.V., The Netherlands) as previously described [24]. The overexpression construct with the same backbone expressing dsRED served as a negative control.

TNS3 site-specific deletions were generated using Q5® Site-Directed Mutagenesis Kit (New England Biolabs, MA, USA) according to the manufacturer’s instructions. Briefly, DNA was amplified using Q5 Hot Start High-Fidelity DNA Polymerase with primers (shown in Table S2) designed by NEBaseChanger™ tool (https://nebasechanger.neb.com). Subsequent products were treated with Kinase-Ligase-DpnI enzyme mix to remove the template. After transformation and *E. coli* cell culture, plasmid DNA was isolated and verified by Sanger sequencing.

As described [24], lentiviruses silencing *TNS3* were packed in HEK293FT cells with ViraPower™ mix (ViraPower Systems, Life Technologies Europe B.V., The Netherlands) using calcium phosphate precipitation-mediated transfection. Lentiviruses overexpressing *TNS3* and its site-specific deletions were produced in HEK293FT cells using Lipofectamine™ 2000 (Life Technologies Europe B.V., The Netherlands) according to the manufacturer’s instructions. BMSCs were transduced with shRNA or overexpression constructs 24 h prior to induction of osteogenic or adipogenic differentiation.

### Alkaline phosphatase (ALP) activity and mineralization assays

ALP activity was measured using the *p*-nitrophenyl phosphate (*p*NPP) method by converting *p*NPP substrate to the equal amount of *p-*Nitrophenol (*p*NP) [24, 26, 27]. Cell lysates were harvested at indicated time points using PBS containing 0.1% triton X-100, followed by the conversion step, which was performed at 37 °C for 10 min. Protein concentration was measured using a BCA protein assay kit (Thermo fisher scientific, Waltham, MA, USA) and further calculated following manufacturer’s instructions. ALP activity was quantified by measuring absorbance at 405 nm and adjusted to protein amount.

Calcium content was determined using a combination of 0.35 mM 0-cresolphthalein with 1 M ethanolamine buffer (pH 10.6) in a 1:1 ratio after incubating the cell lysates and remaining plates overnight with 0.24 M HCl at 4 °C [26]. Calcium concentrations was quantified by measuring absorbance at 595 nm. Total calcium content was calculated by combining calcium in cell lysates and calcium remaining in the plates, and further adjusted with protein amount.

For Alizarin Red S staining, cells were incubated with Alizarin Red S solution (pH 4.2, Sigma-Aldrich, St. Louis, MO, USA) for 15 min at room temperature (RT) after fixation with 70% ethanol, as described previously [26].

### Pyrophosphate assay

Intracellular and extracellular levels of pyrophosphate were determined using a pyrophosphate assay kit according to the manufacturer’s instruction (PromoKine, Huissen, The Netherlands). Cells extracted directly in PPi assay buffer by scraping before proceeding with the measurement. Supernatant from each sample was centrifuged and pre-cleared through a 10 kDa spin column to remove (larger) proteins. After 60-min incubation at 37 °C, samples were measured for their fluorescence at Ex = 535/Em = 587 nm in endpoint mode. Pyrophosphate was calculated using the standard curve and adjusted for protein concentration.

### Oil red O staining

To detect lipid droplets [26], BMSCs were fixed with 10% formalin after 14 days of adipogenic induction, and subsequently incubated for 30 min at RT with Oil Red O solution (Sigma-Aldrich, St. Louis, MO, USA). Nuclei were stained with DAPI to determine cell number. Images (5 images per well) were taken by a Zeiss Axiovert 200MOT microscope (Zeiss, Sliedrecht, The Netherlands) and analyzed using Fiji software. Lipid droplets were extracted with isopropanol and measured absorbance at 490 nm. Normalized absorbance was calculated using raw absorbance divided by cell count.

### RNA isolation, cDNA synthesis, and qPCR

RNA was isolated using TRIzol reagent (Thermo Fisher Scientific, MA, USA) and further processed with cDNA synthesis as described [24–26]. Oligonucleotide primer pairs for qPCR were designed to be either on exon boundaries or spanning at least one intron (Table S3). qPCR was performed on QuantStudio 7 Flex Real-Time PCR system with the GoTaq qPCR Master Mix (Promega, Madison, WI, USA). Gene expression was normalized to the expression of *36B4*, using the Equation: 2^Δ^ − (Ct gene of interest – Ct housekeeping gene).

### Immunocytochemistry

Immunostaining was performed as described [24]. Briefly, cells were fixed with 4% PFA for 5 min and permeabilized using 0.1% Triton X-100 in PBS containing 1% bovine serum albumin (BSA) for 30 min at RT. Cells were incubated with α-tubulin antibody (Cell signaling #2125, Leiden, The Netherlands) or with a-RhoA (NewEast Biosciences; #26,904) at a dilution of 1:100 in PBS containing 0.02% Tween-20 (PBS-T) overnight at 4 °C, followed by secondary antibody incubation with Alexa Fluor 488 donkey anti-rabbit at a dilution of 1:200 (Abcam #150,073, Cambridge, UK) and rhodamine-conjugated phalloidin at a dilution of 1:100 (Invitrogen #10063052, MA, USA) at RT. After washes, images were stained with DAPI and visualized using a confocal laser scanning microscope (Leica Microsystems, Mannheim, Germany).

### Western blot

As described [24], protein samples were prepared using RIPA lysis buffer and separated by SDS-PAGE (4–12% SDS–polyacrylamide gels, Bio-Rad Laboratories, CA, USA). Each membrane was blocked with 5% non-fat milk in TRIS-buffered saline containing 0.1% Tween-20 (TBS-T) after transfer, followed by blotting with primary antibodies overnight at 4 °C. After washes in TBS-T, the membrane was incubated with secondary antibodies for 1 h at RT. The proteins of interest were detected using the Clarity™ Western ECL Substrate (Bio-Rad Laboratories B.V., Veenendaal, The Netherlands) and were quantified using Image Lab software (Bio-Rad Laboratories B.V., Veenendaal, The Netherlands). Antibodies used for Western blot analyses were as follows: TNS3 (1:1,000; Novus Biologicals #NBP2-37,948,, Abingdon, UK), PPARγ (1:1,000; Cell signaling #2435 T, Leiden, The Netherlands), FABP4 (1:1,000; Cell signaling #2120, Leiden, The Netherlands), CEBP/α (1:1,000; Cell signaling #8178, Leiden, The Netherlands), β-Actin (1:1,000; Cell signaling #4970, Leiden, The Netherlands), Anti-rabbit IgG, HRP-linked Antibody (1:2,000; Cell signaling #7074, Leiden, The Netherlands).

### RhoA activation assay

RhoA activity was determined using the RhoA pull-down activation assay kit (Cytoskeleton, Denver, CO, USA). Cells were lysed after washes with PBS following 3 or 12 days of osteogenic induction. Equal amounts of cell lysates were incubated with 50 μg Rhotekin-RBD protein beads for 1 h at 4 °C. After washes, the bound proteins were analyzed by Western blots against an anti-RhoA antibody at a dilution of 1:500 (Cytoskeleton # ARH05, Denver, CO, USA).

### Statistical analysis

All statistical analyses were performed using GraphPad Prism 9. Data were shown as means ± SEM of representative experiments. All experiments were performed at least two times. Significance was analyzed using the unpaired Student’s *t*-test or ANOVA corrected with post hoc testing.

## Results

### TNS3 expression is oppositely regulated after induction of osteogenic and of adipogenic differentiation

**Fig. 1:**
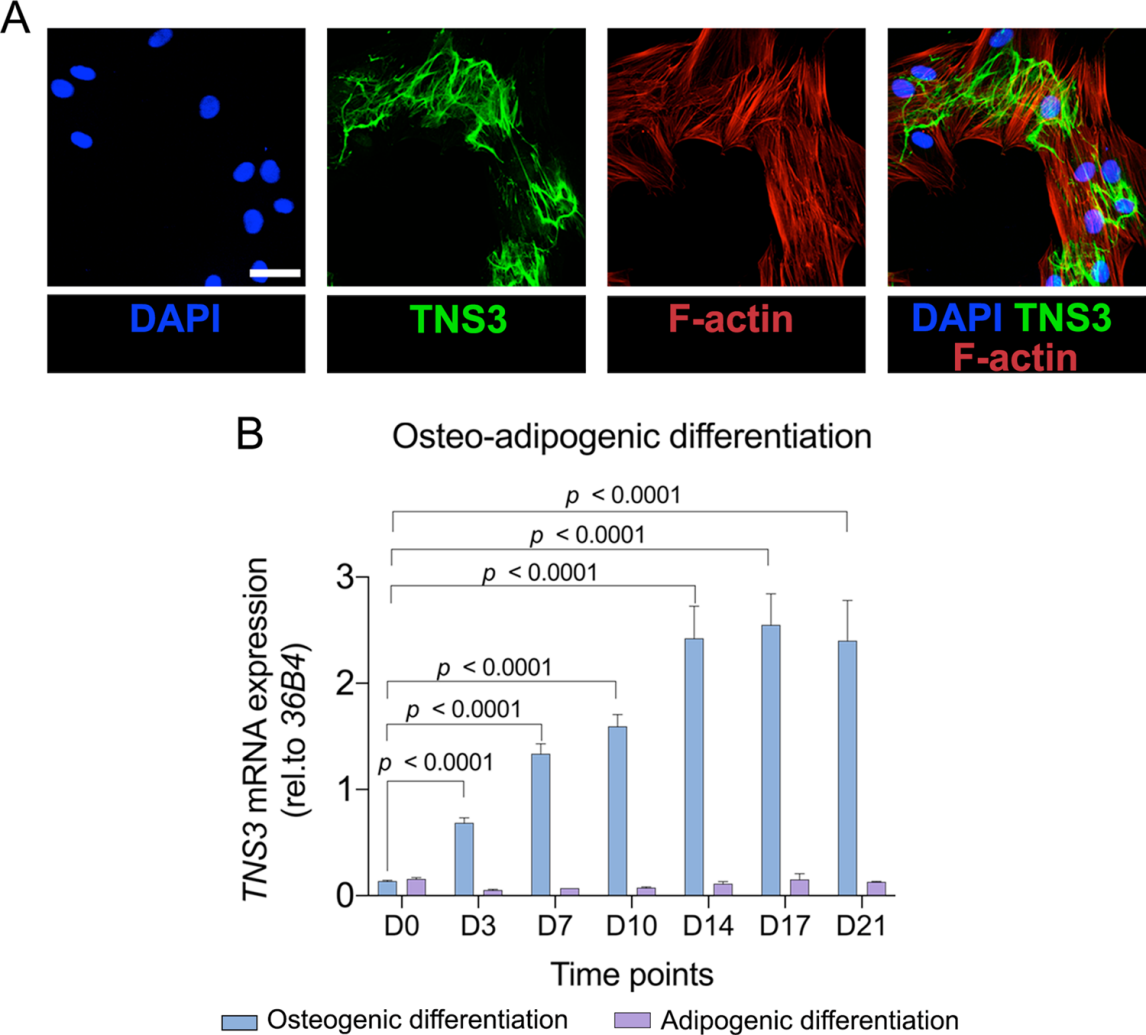
TNS3 is upregulated during osteogenic differentiation and downregulated during adipogenic differentiation. **A** Intracellular localization of TNS3 in BMSCs after 3 days osteogenic induction. Confocal images of immunostainings for TNS3 (Alexa Fluor 488), F-actin (phalloidin-rhodamine) and nuclei (DAPI) were taken. **B** mRNA expression of *TNS3* in BMSCs under osteogenic (blue) and adipogenic conditions (purple). Data (*n* = 2 per group) were presented as means ± SEM and analyzed by two-way ANOVA followed by post hoc testing. Scale bars: 200 µm

The intracellular localization of TNS3 has been shown in tumor cells, but had yet to be determined in BMSCs. To characterize its role during osteogenic differentiation, we immunostained TNS3 in BMSCs after 3 days of osteogenic induction. As shown in Fig. 1A, TNS3 was predominately located in the cytoplasmic region, with no clear co-localization with actin filaments. The expression of *TNS3* was significantly increased after the onset of osteogenic differentiation (versus day 0), peaking between day 17 and day 21, which is the important time frame for calcium phosphate deposition (Fig. 1B). On the other hand, *TNS3* expression remained low after the onset of adipogenic differentiation compared to non-differentiating cells at day 0 (Fig. 1B). These data suggest that TNS3 participates in the differentiation process of BMSCs.

### Silencing TNS3 inhibits osteogenic differentiation and mineralization

**Fig. 2:**
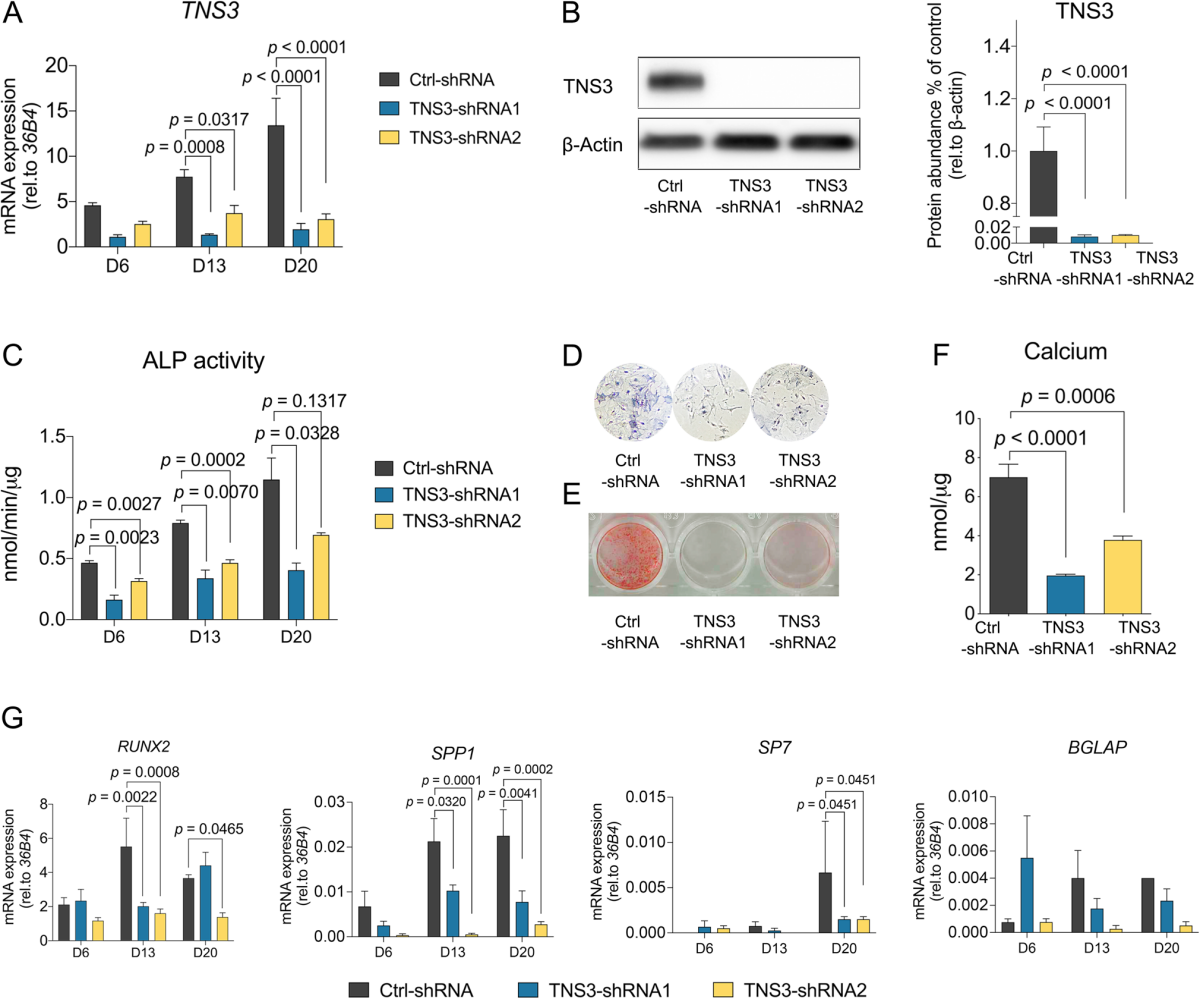
Silencing *TNS3* inhibits osteogenic differentiation and mineralization in BMSCs. **A–B** The mRNA (**A**) and protein (**B**) level of TNS3 transduced with control, and shRNA1 and shRNA2 under osteogenic inductions. mRNA expression of *TNS3* was measured at indicated time points. TNS3 protein expression was evaluated after 3 days of osteogenic induction. BMSCs were transduced with shRNA constructs 24 h prior to induction of osteogenic differentiation. **C** ALP activity was evaluated at different time points under osteogenic induction. **D** ALP staining was performed at day 13 under osteogenic induction. **E–F** Osteoblast mineralization was assessed by Alizarin Red S staining (**E**) and calcium deposition assay (**F**) following 3 weeks of osteogenic induction. **G** qRT-PCR for *RUNX2, SPP1, SP7,* and *BGLAP* mRNA expression at indicated time points during osteogenesis. All data were presented as means ± SEM, and analyzed by one-way (**B** and **F**, *n* = 3–4 per group) or two-way (**A**, **C,** and **G, ***n* = 4 per group) ANOVA followed by post hoc testing

To explore the role of TNS3 during osteogenic differentiation, we knocked down *TNS3* with short hairpin RNAs (shRNAs), using lentiviral-mediated transduction. *TNS3* silencing was achieved by two different shRNAs at the mRNA level (Fig. 2A), and the knockdown efficiency was further confirmed by Western blotting (Fig. 2B). Inactivation of TNS3 notably inhibited osteogenic differentiation, as shown by reduced ALP activity and ALP staining (Fig. 2C). Calcium deposition, the phenotypic marker for extracellular matrix mineralization, was decreased following *TNS3* silencing (Fig. 2E–F). Furthermore, the suppressed osteogenesis was underlined by decreased expression of *RUNX2*, *SPP1* and *SP7* in the *TNS3* knockdown conditions (Fig. 2G). A trend for decreased *BGLAP* expression was also observed with both shRNAs at the late time point (Fig. 2G). These observations suggest that TNS3 is critical for osteogenic differentiation and mineralization.

### Silencing TNS3 enhances adipogenic differentiation of BMSCs

**Fig. 3:**
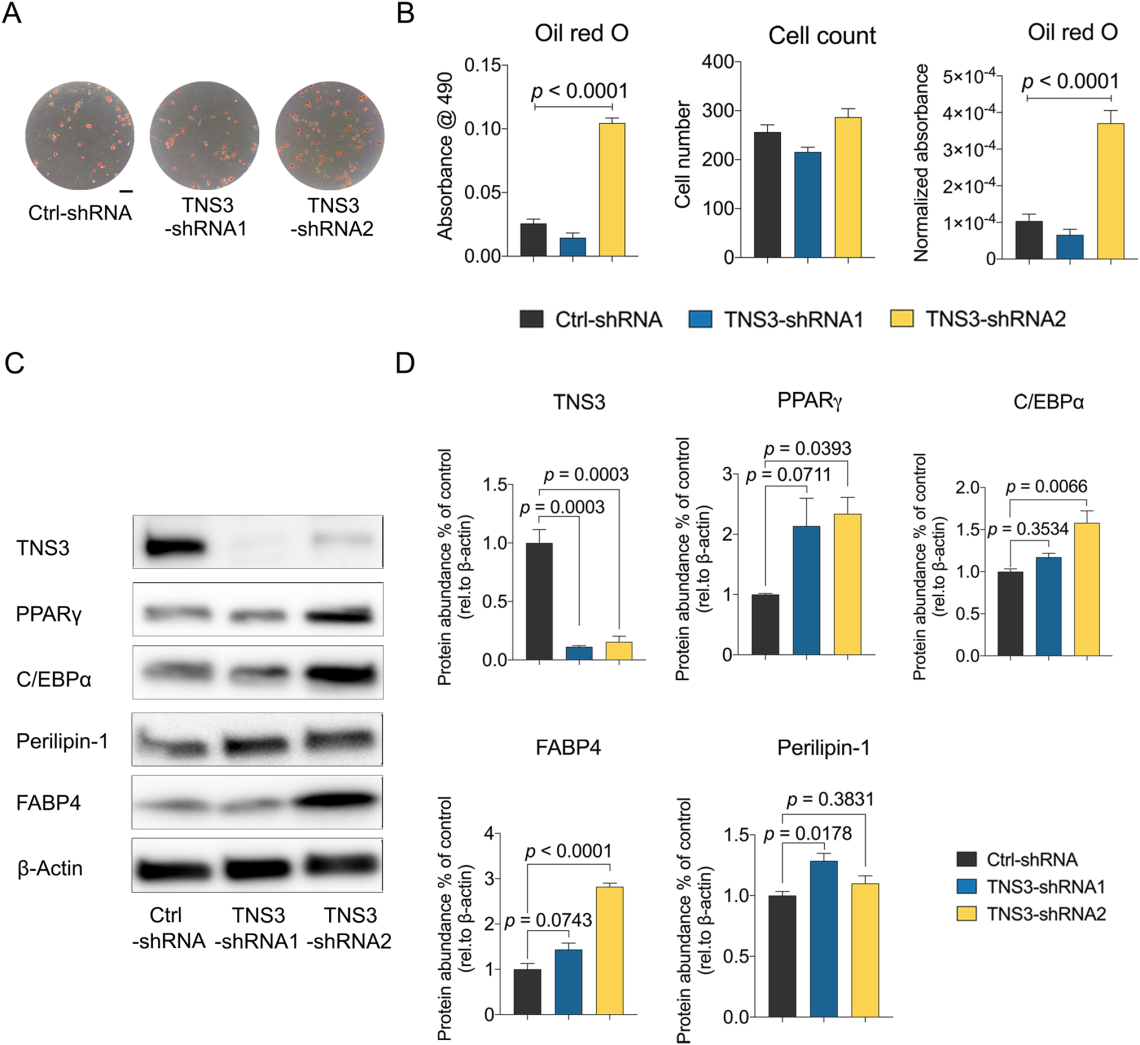
Silencing *TNS3* enhances adipogenic differentiation in BMSCs.** A–B** Representative images (**A**) and quantitative data (**B**) of Oil Red O staining on BMSCs after being cultured 14 days in adipogenic condition. Cell number was determined by DAPI staining. Quantitative data of Oil Red O staining was adjusted by cell count and indicated as normalized absorbance. **C–D** Representative images (**C**) and quantitative data (**D**) of adipogenic markers measurements after 7 days adipogenic induction by western blotting. BMSCs were transduced with shRNA constructs 24 h prior to induction of adipogenic differentiation. Data were presented as means ± SEM and analyzed by one-way ANOVA (**B** and **D,** *n* = 3–4 per group) followed by post hoc testing. Scale bars: 100 µm

A number of studies have demonstrated that the lineage commitment process of BMSC towards adipocytes requires the inhibition of osteogenesis [7, 28]. Due to the observation that knockdown of *TNS3* reduces the osteogenic capacity, we sought to assess whether loss of TNS3 would affect adipocyte differentiation. To achieve this, we assessed lipid droplet formation and adipogenic marker expression in *TNS3* silencing condition. As shown in Fig. 3A and B, the ablation of *TNS3* with shRNA2 led to a drastic increase in Oil Red O staining at 14 days following adipogenic induction, while shRNA1 did not. The marked increase of the staining was not caused by the difference in cell proliferation because cell number was not altered when treating with the shRNAs. Although the protein levels for the adipogenic markers PPARG, FABP4 (both not significant) and PLIN1 (significant) showed an elevated trend for shRNA1 as well, only the effects of shRNA2 on Oil Red O staining were supported by significant changes of the adipogenic markers at protein (Fig. 3C–D) and mRNA level (Fig. S1) post *TNS3* silencing. Collectively, these data suggest that the downregulation of *TNS3* facilitates BMSCs to become adipocytes.

### TNS3 overexpression oppositely regulates osteogenesis and adipogenesis in BMSCs

**Fig. 4:**
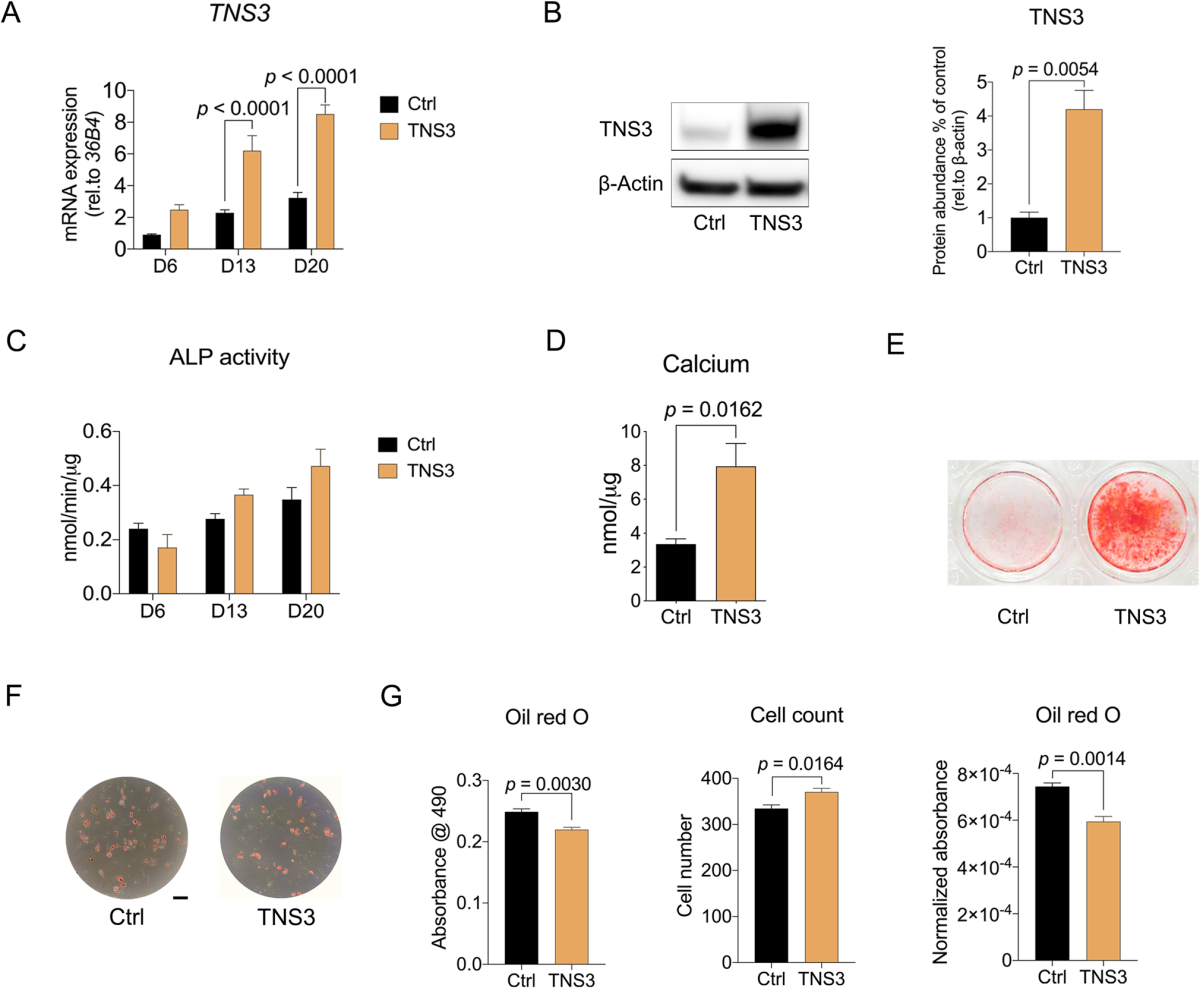
*TNS3* overexpression oppositely regulates osteogenesis and adipogenesis in BMSCs. **A–B** The mRNA (**A**) and protein (**B**) level of TNS3 transduced with control, and TNS3 lentiviral construct under osteogenic inductions. mRNA expression of *TNS3* was measured at indicated time points. TNS3 protein expression was evaluated after 3 days of osteogenic induction. **C** ALP activity was evaluated at different time points under osteogenic induction. **D–E** Osteoblast mineralization was evaluated by calcium deposition assay (**D**) and Alizarin Red S staining (**E**) after 3-week osteogenic induction. **F–G** Representative images (**F**) and quantitative data (**G**) of Oil Red O staining on BMSCs after 14 days adipogenic differentiation. Cell number was determined by DAPI staining. Quantitative data of Oil Red O staining was adjusted by cell count and indicated as normalized absorbance. All data were presented as means ± SEM, and analyzed by two-sided Student’s *t*-test (**B, D,** and **G**, *n* = 3–4 per group) or two-way ANOVA (**A and C**, *n* = 4 per group) followed by post hoc testing

To further depict the role of TNS3 in BMSC differentiation, we overexpressed *TNS3* and assessed the capabilities of BMSCs to differentiate into the osteogenic and adipogenic pathway. Lentiviral-mediated transduction dramatically increased *TNS3* mRNA expression measured at different time points (Fig. 4A), which was confirmed at protein level (Fig. 4B). During osteogenesis, ALP activity was increased from day 13 onward in response to *TNS3* overexpression, albeit not significant (Fig. 4C). Elevated mineral deposition was observed quantitatively by measurement of calcium deposition (Fig. 4D), and confirmed qualitatively by Alizarin Red S staining (Fig. 4E).

In contrast to the stimulatory effect on osteogenic differentiation, overexpression of *TNS3* in BMSCs suppressed adipocyte proliferation evaluated by cell number, and more importantly, the lineage commitment process towards adipocytes, as revealed by Oil Red O staining (Fig. 4F–G). Together, these data reinforce our hypothesis that TNS3 stimulates osteogenesis but is inhibitory to adipogenesis.

### TNS3 enhances osteogenic differentiation through its domain functions

**Fig. 5:**
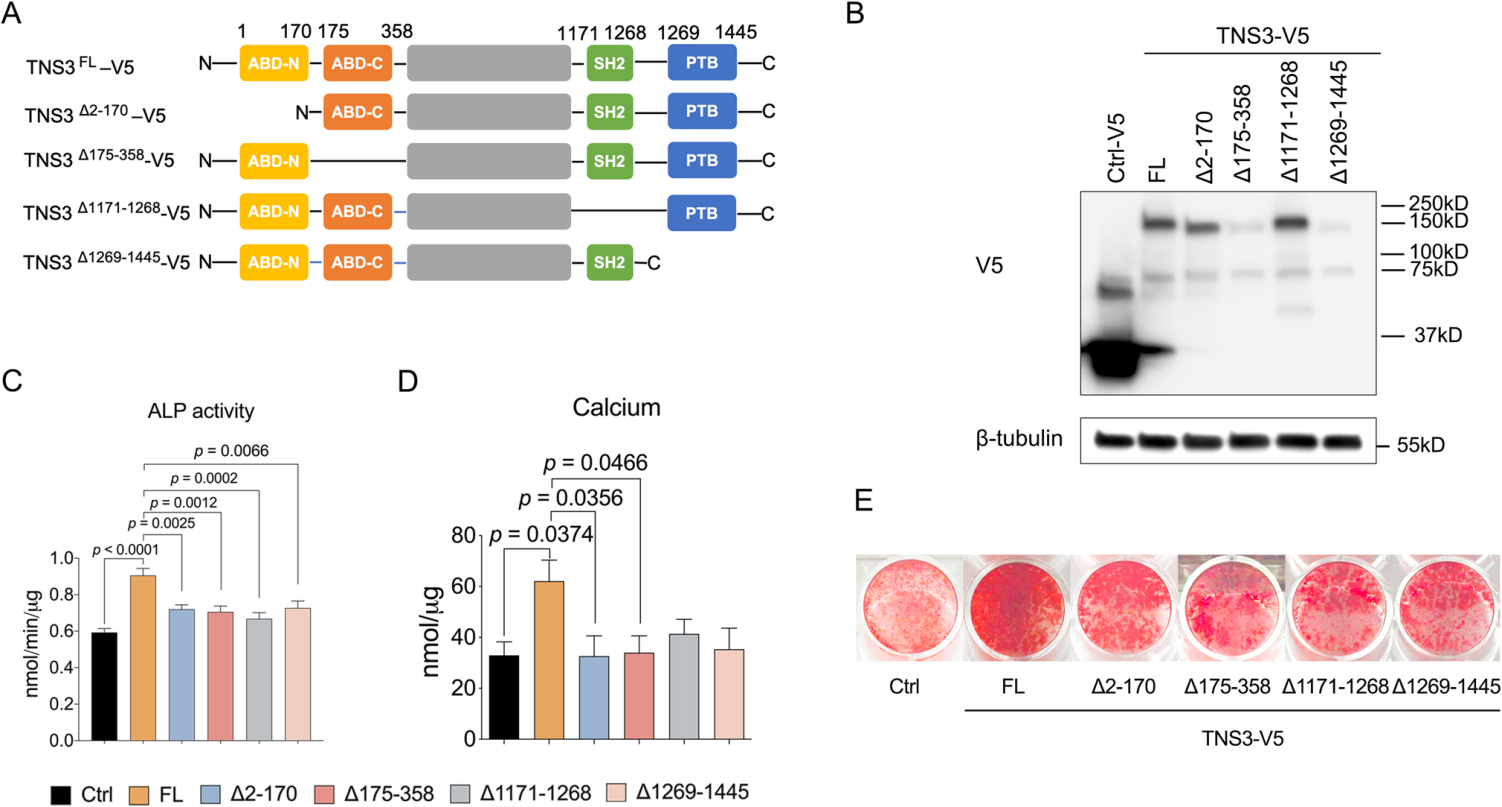
The ability of TNS3 to enhance osteogenic differentiation depends on the N- and C-terminal actin-binding domains.** A** Schematic representation of TNS3 truncated mutants. **B** BMSCs expressing the indicated deletion constructs were immunoblotted with anti-V5 antibody in non-differentiating conditions. **C** ALP activity was evaluated in BMSCs expressing indicated constructs after 6 days osteogenic induction. **D–E** Osteoblast mineralization was assessed by calcium deposition assay (**D**) and Alizarin Red S staining (**E**) after being cultured under osteogenic induction for 3 weeks. All data (*n* = 4 per group) were presented as means ± SEM and analyzed by one-way ANOVA followed by post hoc testing. Abbreviation: FL, full-length

TNS3 mediating signal transduction and physiological processes heavily depends on its structural domains [17]. Therefore, we examined the biological roles of TNS3 domains in osteogenic differentiation of BMSCs using various deletion mutants (Fig. 5A). BMSCs overexpressing the different TNS3 deletion constructs were immunoblotted with anti-V5 antibody as shown in Fig. 5B. Compared to the full-length overexpression condition, ALP activity was reduced when overexpressing each of the four deletion constructs (Fig. 5C). Calcium deposition assays revealed that the expression of TNS3 with a deletion of its ABD-N domain (N-terminal actin-binding domain, Δ2–170) or ABD-C domain (C-terminal actin-binding domain, Δ175–358) blunted calcium deposition in mineralized cultures (Fig. 5D), which was further confirmed by Alizarin Red S staining (Fig. 5E). On the other hand, deletion of the SH2 domain (Src homology 2 domain, Δ1171–1268) or PTB domain (phosphotyrosine-binding domain, Δ1269–1445) also showed a strong reduction in mineralization but this did not reach significance (*p* = 0.0615, Fig. 5D–E).

To determine whether impaired mineralization was caused by altered subcellular localization of deletion mutants, we performed immunostainings, using V5 antibody to detect full-length and truncated TNS3 proteins. As shown in Supplementary Fig. 2, all four deletion mutants had similar subcellular localization as full-length TNS3. Consistent with the findings from the Western blot (Fig. 5B), lacking the ABD-N or PTB domain in TNS3 suppressed protein expression compared to full-length TNS3 (Fig. S2), which was not observed when assessing their mRNA expression (Fig. S3). Overall, deletion of any of the TNS3 domains leads to impaired osteoblast differentiation.

### TNS3 expression in BMSCs affects cytoskeleton reorganization

**Fig. 6:**
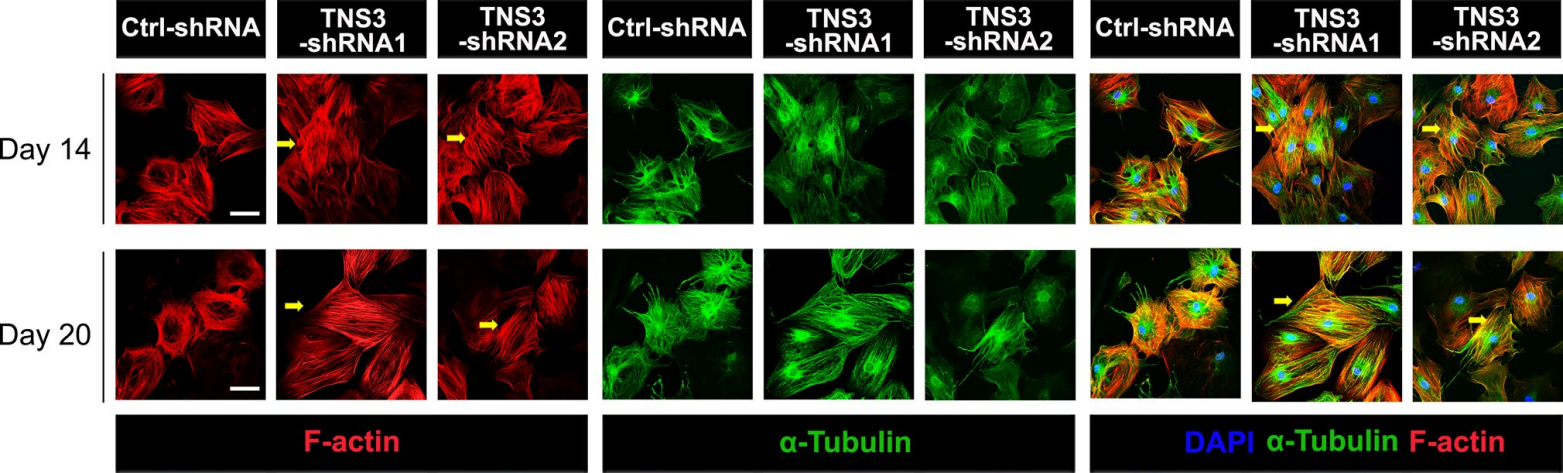
*TNS3* silencing in BMSCs affects cytoskeleton reorganization. Confocal images of immunostainings for F-actin (phalloidin-rhodamine), α-tubulin (Alexa Fluor 488) and nuclei (DAPI) following 14- or 20-day osteogenic induction. Arrows indicate actin filaments across the entire cytoplasm. Scale bars: 200 μm

Morphological changes in cells and extensive modifications of the cytoskeleton are important for BMSCs differentiation into osteoblasts. To determine whether these dynamic changes are influenced by TNS3, we immunostained BMSCs for F-actin and α-tubulin at days 3, 7, 14, and 20. During osteogenic induction under control conditions, BMSCs underwent progressive morphological changes, which was accompanied by actin rearrangement displayed from parallel filaments traversing the entire cell body to filament bundles located around the outermost parts of the cell and end up with the star shaped osteoblast like morphology (Fig. 6 and Fig. S4). In contrast, delayed actin reorganization was visualized from 14 days of osteogenic induction onwards and this observation was most evident at day 20 following *TNS3* silencing (Fig. 6), while α-tubulin staining did not show distinguishable morphological differences compared to the control condition (Fig. 6).

**Fig. 7:**
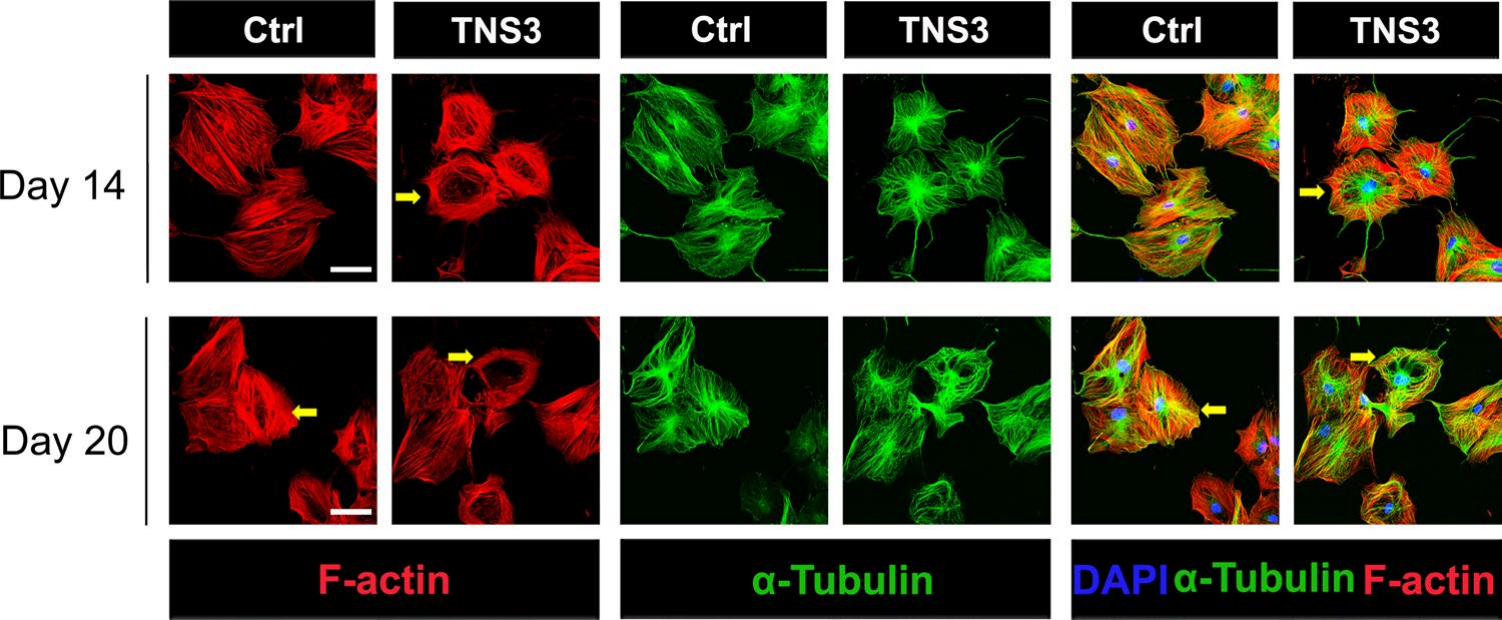
Overexpression of *TNS3* in BMSCs influences cytoskeleton reorganization. Confocal images of immunostainings for F-actin (phalloidin-rhodamine), α-tubulin (Alexa Fluor 488), and nuclei (DAPI) following 14- or 20-day osteogenic induction. Arrows indicate star shaped mature osteoblasts. Scale bars: 200 μm

Remarkably, immunostaining revealed the accelerated reorganization of actin filaments in the *TNS3* overexpression condition compared to control following 14 days osteogenic induction (Fig. 7), while the difference was less obvious at day 20 (Fig. 7). Collectively, these data demonstrated the expression of TNS3 affect actin rearrangement during osteogenic differentiation.

### Silencing TNS3 in BMSCs has no effect on the levels of pyrophosphate (PPi)

**Fig. 8:**
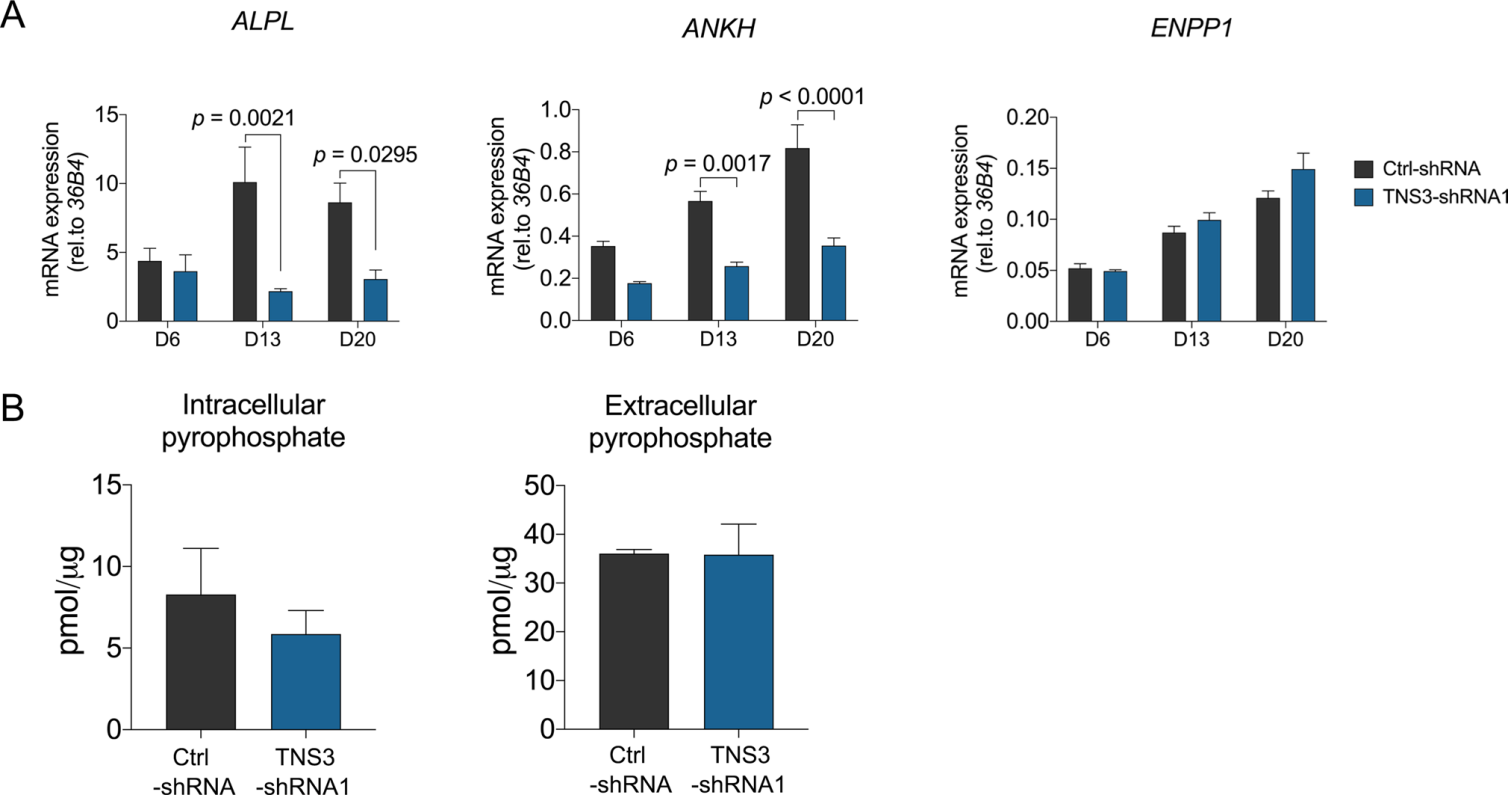
Silencing *TNS3* in BMSCs has no effect on the levels of pyrophosphate (PPi) in BMSCs.** A** mRNA expression of *ALPL*, *ANKH* and *ENPP1* were measured at indicated time points during osteogenic differentiation after *TNS3* silencing with shRNA1. **B** Intracellular PPi and extracellular PPi were evaluated after being cultured in osteogenic medium for 3 days following knockdown of *TNS3*. Intracellular PPi and extracellular PPi were measured in cell lysates and supernatant, respectively. All data were presented as means ± SEM, and analyzed by two-way ANOVA (**A**, *n* = 4 per group) followed by post hoc testing or two-sided Student’s *t*-test (**B**, *n* = 3–4 per group)

Ample studies have shown that ALP function is essential for skeletal mineralization through regulating the hydrolyzation of PPi, which is an inhibitor of hydroxyapatite crystal growth [29, 30]. The reduced ALP activity and *ALPL* expression (Fig. 2C and Fig. 8A, respectively) triggered us to explore whether the level of PPi was affected by changes in *TNS3*. ShRNA1 was selected for further studies to investigate the role of TNS3 as it showed the strongest inhibition of osteogenesis. The generation of extracellular PPi occurs either through the breakdown of nucleotide triphosphates catalyzed by ectonucleotide pyrophosphatase (ENPP1), or through the export from intracellular region operated by ANK transporter (ANKH). As shown in Fig. 8A, the expression of *ENPP1* was slightly elevated with *TNS3* silencing (not significant), which could potentially lead to increased extracellular PPi level. On the other hand, the transporter gene *ANKH* was strongly downregulated in response to *TNS3* knockdown (Fig. 8A), leading to reduced export of PPi from the intracellular environment. However, there are no obvious changes in intracellular and extracellular levels of PPi following *TNS3* knockdown (Fig. 8B). Collectively, these data indicate that *TNS3* silencing changed the expression profile of PPi-related genes, but the levels of PPi production remained stable.

### TNS3 regulates osteogenic differentiation by mediating RhoA-GTP

**Fig. 9:**
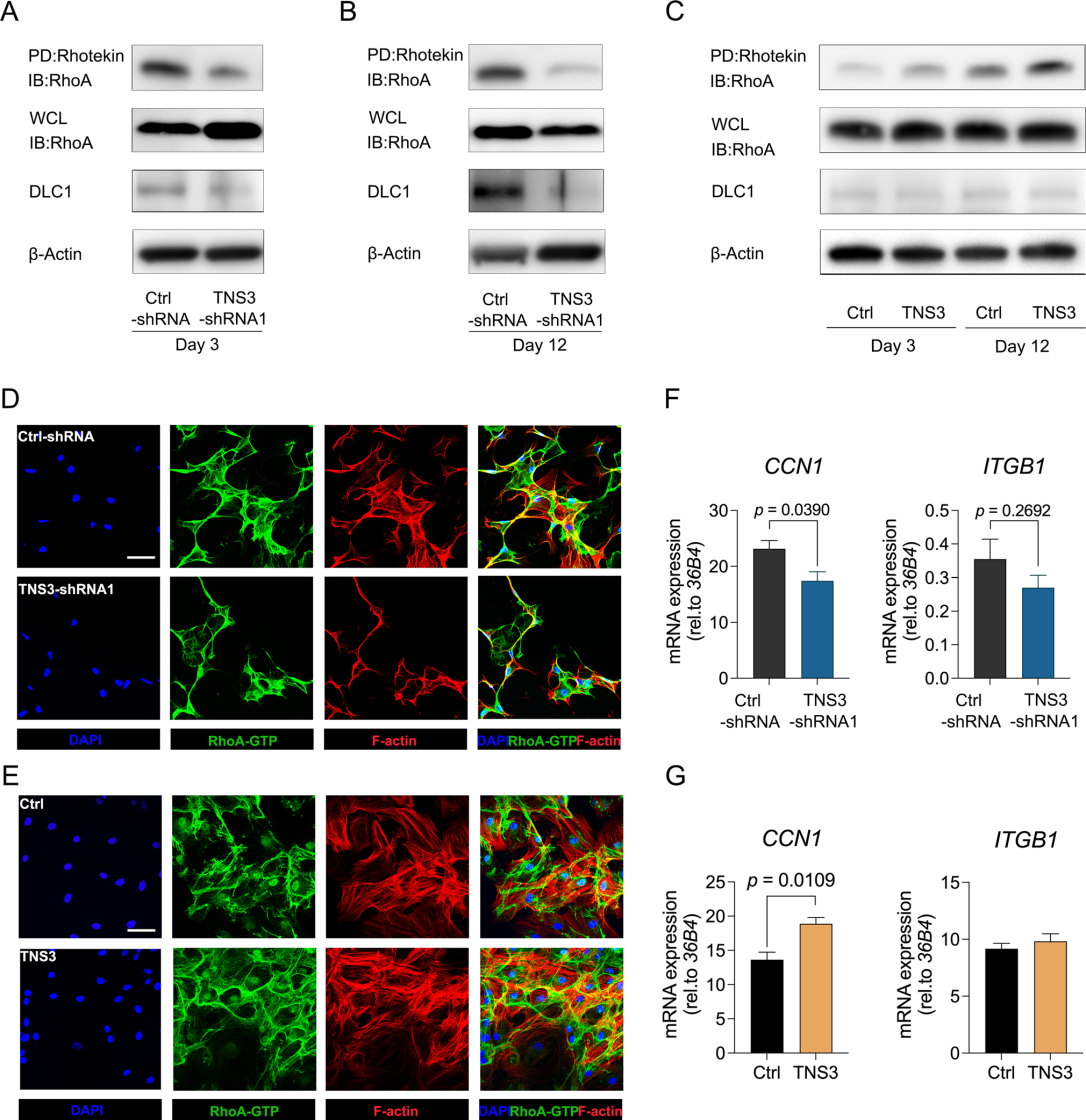
TNS3 regulates osteogenic differentiation by mediating RhoA-GTP. **A–C** The levels of RhoA and DLC1 were assessed in BMSCs transduced with *TNS3* silencing shRNA1. **A**–**B** or *TNS3* overexpression construct. **C** in osteogenic condition at day 3 and day 12. Active RhoA was evaluated by Rhotekin pull-down followed by anti-RhoA immunoblotting. Abbreviation: PD, pull-down, IB, immunoblot, whole cell lysates (WCL). **D–E** Confocal images of immunostainings for F-actin (phalloidin-rhodamine), RhoA-GTP (Alexa Fluor 488) and nuclei (DAPI) following 12 days of osteogenic induction. **F–G** The levels of *CCN1* and *ITGB1* were assessed in BMSCs in the presence of osteogenic induction at day 6

RhoA is one of the best characterized small GTPases from the Rho family with essential roles in the regulation of cytoskeletal organization through the assembly of actin filaments, thereby affecting cell differentiation [31–33]. In keeping with the observed cytoskeleton changes, we hypothesized that TNS3 regulates osteogenesis through changes in RhoA activity. The level of RhoA-GTP was reduced at the early stage of osteogenic differentiation following *TNS3* silencing, even with the strong expression of total RhoA (Fig. 9A). This decrease was consistent with that observed at 12 days (Fig. 9B). DLC1, which has been previously demonstrated to bind TNS3 [18], showed a decreased expression at both early and late stages of osteogenic differentiation in following *TNS3* silencing (Fig. 9A–B). In addition, overexpression of *TNS3* continually increased the level of RhoA-GTP in osteogenic condition, without eliciting significant changes for DLC1 (Fig. 9C). In corroboration with the RhoA-GTP protein levels following TNS3 silencing and overexpression, RhoA co-localization with the actin cytoskeleton at day 12 of osteogenic differentiation appeared to be reduced following TNS3 silencing, while it was elevated when TNS3 is overexpressed (Fig. 9D and E, respectively). This effect was not yet visible at day 3 of differentiation (Fig.S5). Further mechanistic insights focusing on downstream targets of TNS3 come from assessing the gene expression of the ECM gene cellular communication network 1 (CCN1; a.k.a. Cyr61) and integrin β1 (ITGB1). CCN1 expression was significantly down- or upregulated following TNS3 silencing or overexpression, while ITGB1 only seemed to be downregulated following TNS silencing, albeit non-significantly. Taken together, these results demonstrated that RhoA localization in the cytoskeleton is involved in osteogenic differentiation mediated by TNS3.

## Discussion

Our results demonstrated that TNS3 plays a critical role in regulating BMSCs differentiation. The expression of TNS3 is positively associated with osteogenic differentiation and mineralization, and conversely, negatively correlated with adipogenic differentiation and adipocyte formation using gain- and loss-of-function models in BMSCs. TNS3 domain deletion studies showed that all studied domains were essential for optimal TNS3-mediated osteogenic differentiation. Mechanistically, we found cell shape and actin filaments to go through dramatic changes during osteogenic differentiation, which were disrupted or accelerated by silencing or overexpression of *TNS3*, respectively. These morphologic changes triggered progressive and sustained alteration of RhoA activity, further affecting osteogenesis in BMSCs. These data support a role for TNS3 as a critical regulator in BMSC-derived osteogenic and adipogenic differentiation.

### TNS3 is involved in osteogenesis and adipogenesis

The family of tensins has been implicated in tumorigenesis where it was originally discovered as an actin-binding component of the focal adhesion complex [34]. Embryos derived from *Tns3* mutant mice were significantly smaller than wild-type mice due to reduced longitudinal growth [21]. Together with the expression pattern that *TNS3* is upregulated under osteogenic induction triggered us to investigate its role on the differentiation of BMSCs. Using knockdown and overexpression methods on BMSC, the current study revealed that TNS3 intrinsically promotes osteogenesis, consistent with a previous report using tonsil-derived BMSCs [35]. As BMSC fate towards osteoblasts versus adipocytes is interrelated, we wondered whether the adipogenic differentiation of BMSCs could be affected by TNS3 interference. In difference with the observation that both shRNAs showed similar inhibitory effects on osteogenic differentiation, *TNS3* silencing with shRNA2 led to a drastic increase in adipocyte formation, while this effect was not observed using shRNA1. We do not have a good explanation for this discrepancy and perhaps a later time point would have revealed similar outcomes as for shRNA2, but we did observe a trend towards increased protein levels for PPARG and FABP4 and a significantly increased level of PLIN1 following shRNA1-mediated *TNS3* silencing. Moreover, and in line with the phenotypic effect of *TNS3* shRNA2, overexpression of *TNS3* clearly suppressed the lineage commitment process towards adipocytes, indicating that TNS3 is oppositely involved in osteogenic versus adipogenic differentiation from BMSCs.

Although the mechanisms behind PPi metabolism is not well established, its inhibitory role for mineralization has been confirmed by numerous studies [29, 30]. The reduced ALP activity and increased *ANKH* expression lead us to expect increased extracellular PPi, which could contribute to the inhibition of extracellular mineralization observed following *TNS3* knockdown. However, both intracellular PPi and extracellular PPi remained stable in the *TNS3* silencing condition. This observation could be explained by the fact that the generation of PPi mostly depends on the PPi-synthesizing activity of ENPP1 than on the PPi transport function of ANKH [36]. Indeed, we did not see significant changes in the expression of *ENPP1*, suggesting a limited role for *TNS3* silencing in PPi metabolism.

### TNS3 is involved in the regulation of the actin cytoskeleton

Previous studies documented that cytoskeletal adaptation contributes to MSC differentiation through mechanical stress- or chemical signal-mediated actin reorganization, which is considered as a pre-requisite for MSC differentiation into osteoblasts [37–40]. The expression level of TNS3 influenced F-actin rearrangement and morphological changes of BMSCs during osteogenic differentiation. It has been reported that integrins, which respond to various mechanical stimuli, can be regulated by TNS3 [41]. Therefore, changes in the TNS3 level may disrupt integrin activities and subsequently suppress mechanical sensing and MSC differentiation. On the other hand, tensins are large proteins consisting of a characteristic set of domains involved in discrete activities through protein–protein interactions, potentially rendering them dysfunctional in case of partial or complete loss of TNS3 domains. Tensins 1–3 bind directly to the actin cytoskeleton through ABDs and connect them to integrin receptors and other associated proteins [16, 17]. Impairment of tensins could, thus, impair the anchorage of actin filaments to the plasma membrane and consequently alter the cytoskeletal structure, resulting in cell shape changes and disturbed capacity of osteogenic differentiation. In agreement with these speculations, we found that deletion of ABDs abolish the stimulatory effect of full-length TNS3 on osteogenesis.

RhoA is an important member of small GTPases from Rho family (Rho-GTPases), and is a major regulator of cell shape changes and actin dynamics [33, 37]. RhoA switches between an active GTP- and inactive GDP-bound state, and which are regulated by guanine nucleotide exchange factors (GEFs) and GTPase activating proteins (GAPs), respectively. The spatial and temporal increase of RhoA-GTP during osteogenic differentiation is in line with the finding that activation of RhoA contributes to osteogenesis [33]. Consistent with previous findings [42], we observed that RhoA-GTP is upregulated by *TNS3* gain-of-function experiments and seems to co-localize with the actin cytoskeleton. In line with the effect of *TNS3* silencing on osteoblast differentiation, we found that *TNS3* silencing suppresses the expression of active RhoA as well as reduced alignment with the cytoskeleton. A study using a breast cancer cell line suggests that *TNS3* silencing activates the Rho-GAP function of DLC1 through releasing an autoinhibitory effect, and thereby elevating the level of RhoA-GTP [18]. However, in agreement with our findings, another study showed that *TNS3* silencing reduces the level of RhoA-GTP in human foreskin cells [43], suggesting that TNS3 regulates RhoA activity in a cell-type-dependent manner. Moreover, silencing *TNS3* leads to a downregulation of DLC1, proposing the dual regulation of DLC1 by TNS3. Although future studies are required to scrutinize the downstream events of TNS3 and RhoA signaling, some of our findings suggest that ITGB1 and CCN1 may be among their targets, which has been suggested previously [35, 44].

## Conclusions

We found TNS3 to regulate the fate of BMSCs towards osteoblasts versus adipocytes, suggesting a role in maintaining a healthy osteo-adipogenic balance. The stimulatory effect of TNS3 on osteogenic differentiation is closely related to morphological changes and cytoskeleton rearrangements. These findings provide new insights into the development of therapeutic modalities to treat metabolic bone diseases and pathogenic statuses such as low bone density, high bone marrow fat and increased fracture risk.

### Supplementary Information

Below is the link to the electronic supplementary material.

Supplementary file1 (TIF 18348 KB) Fig.S1 TNS3 silencing promotes the expression of adipogenesis genes. mRNA expression of adipogenesis genes were measured at indicated time points after adipogenic induction. All data were presented as means ± SEM and analyzed by two-way ANOVA followed by post hoc testing (n = 4 per group)

Supplementary file2 (TIF 31486 KB) Fig.S2 Overexpression of TNS3 deletion mutants have similar intracellular localization as full-length TNS3. hMSCs expressing the indicated deletion constructs were immunostained with V5 (Alexa Fluor 488) and F-actin (phalloidin-rhodamine), and Nuclei (DAPI) after 3 days osteogenic induction. Scale bars: 200 μm

Supplementary file3 (TIF 4142 KB) Fig.S3 The expression levels of TNS3 in deletion constructs. mRNA expression of TNS3 deletion constructs were assessed with qRT-PCR at day 3 using primers targeting TNS3-5’UTR (untranslated region), V5, TNS3.

Supplementary file4 (TIF 16519 KB) Fig.S4 TNS3 silencing in hMSCs affects cytoskeleton reorganization. A-C Confocal images of immunostainings against F-actin (phalloidin-rhodamine), α-tubulin (Alexa Fluor 488) and Nuclei (DAPI) after 3 days and 7 days following osteogenic induction. Scale bars: 200 μm

Supplementary file5 (TIF 36148 KB) Fig.S5 TNS3 does not alter the co-localization of RhoA with the actin cytoskeleton. A-B Confocal images of immunostainings against F-actin (phalloidin-rhodamine), RhoA (Alexa Fluor 488) and Nuclei (DAPI) at 3 days after osteogenic induction following RNA silencing (A) or overexpression (B). Scale bars: 200 μm

Supplementary file6 (DOCX 33 KB)

Supplementary file7 (DOCX 15 KB)

## Data Availability

The datasets generated and analyzed during the current study are available from the corresponding author on reasonable request.

## Abbreviations

**BMSCs**: Bone marrow-derived stromal cells
**RUNX2**: Runt-related transcription factor 2
**C/EBPs**: CCAAT/enhancer binding proteins
**PPAR-γ**: Peroxisome proliferator-activated receptor gamma
**ACBP**: Acyl-CoA-binding protein
**FASN**: Fatty acid synthase
**CTEN**: C-terminal tensin-like
**PTB domain**: Phosphotyrosine-binding domain
**shRNA**: Short hairpin RNAs
**PBS**: Phosphate-buffered saline
**RT**: Room temperature
**ALP**: Alkaline phosphatase
***pNPP***: P-nitrophenyl phosphate
***pNP***: P-Nitrophenol
**BSA**: Bovine serum albumin
**FABP4**: Fatty acid-binding Protein 4
**ABD-N domain**: N-terminal actin-binding domain
**ABD-C domain**: C-terminal actin-binding domain
**SH2 domain**: Src homology 2 domain
**PPi**: Pyrophosphate
**ENPP1**: Ectonucleotide pyrophosphatase/phosphodiesterase 1
**ANKH**: ANKH Inorganic pyrphosphate transport regulator
**Rho-GTPases**: Small GTPases from Rho family
**GEFs**: Guanine nucleotide exchange factors
**GAPs**: GTPase activating proteins
