# Supplementary figures and images for: Tensin-3 is involved in osteogenic versus adipogenic fate of human bone marrow stromal cells
Source: Cell Mol Life Sci. 2023 Sep 5;80(9):277. doi: 10.1007/s00018-023-04930-5 (PMC10480249; doi:10.1007/s00018-023-04930-5)

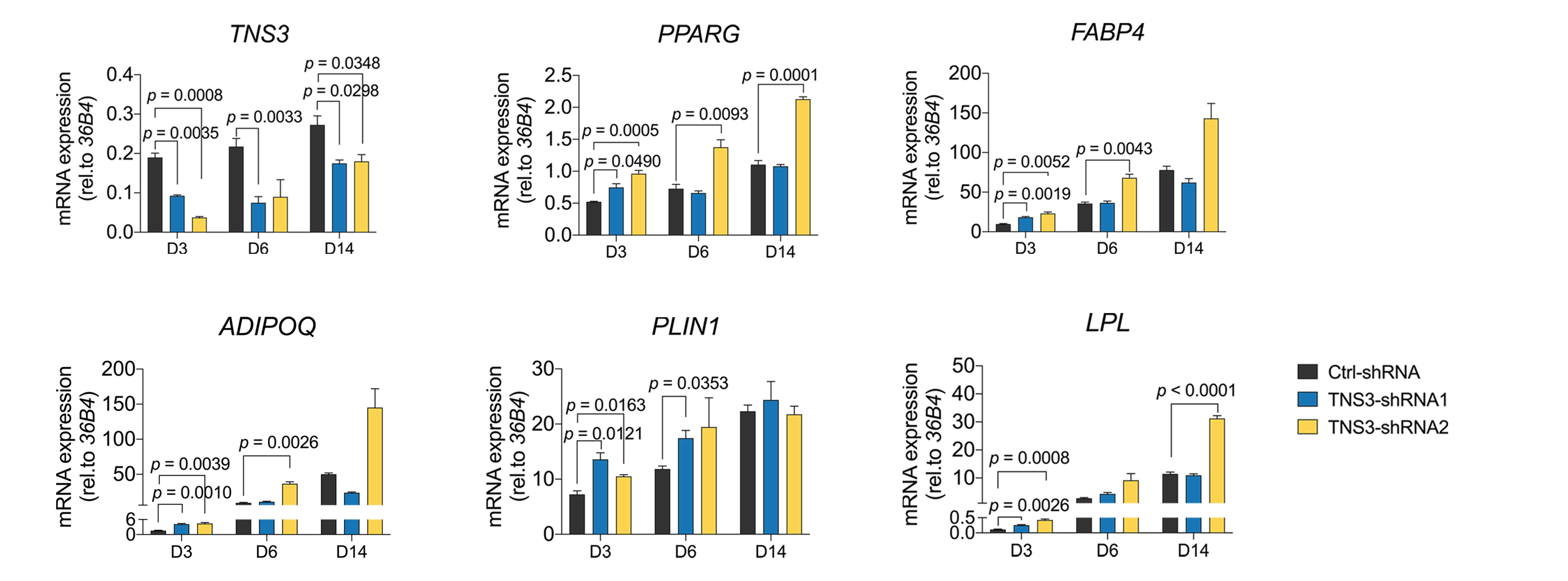

Supplement: Supplementary file 1 — Supplementary file1 (TIF 18348 KB) Fig.S1 TNS3 silencing promotes the expression of adipogenesis genes. mRNA expression of adipogenesis genes were measured at indicated time points after adipogenic induction. All data were presented as means ± SEM and analyzed by two-way ANOVA followed by post hoc testing (n = 4 per group) [file 18_2023_4930_MOESM1_ESM.tif]

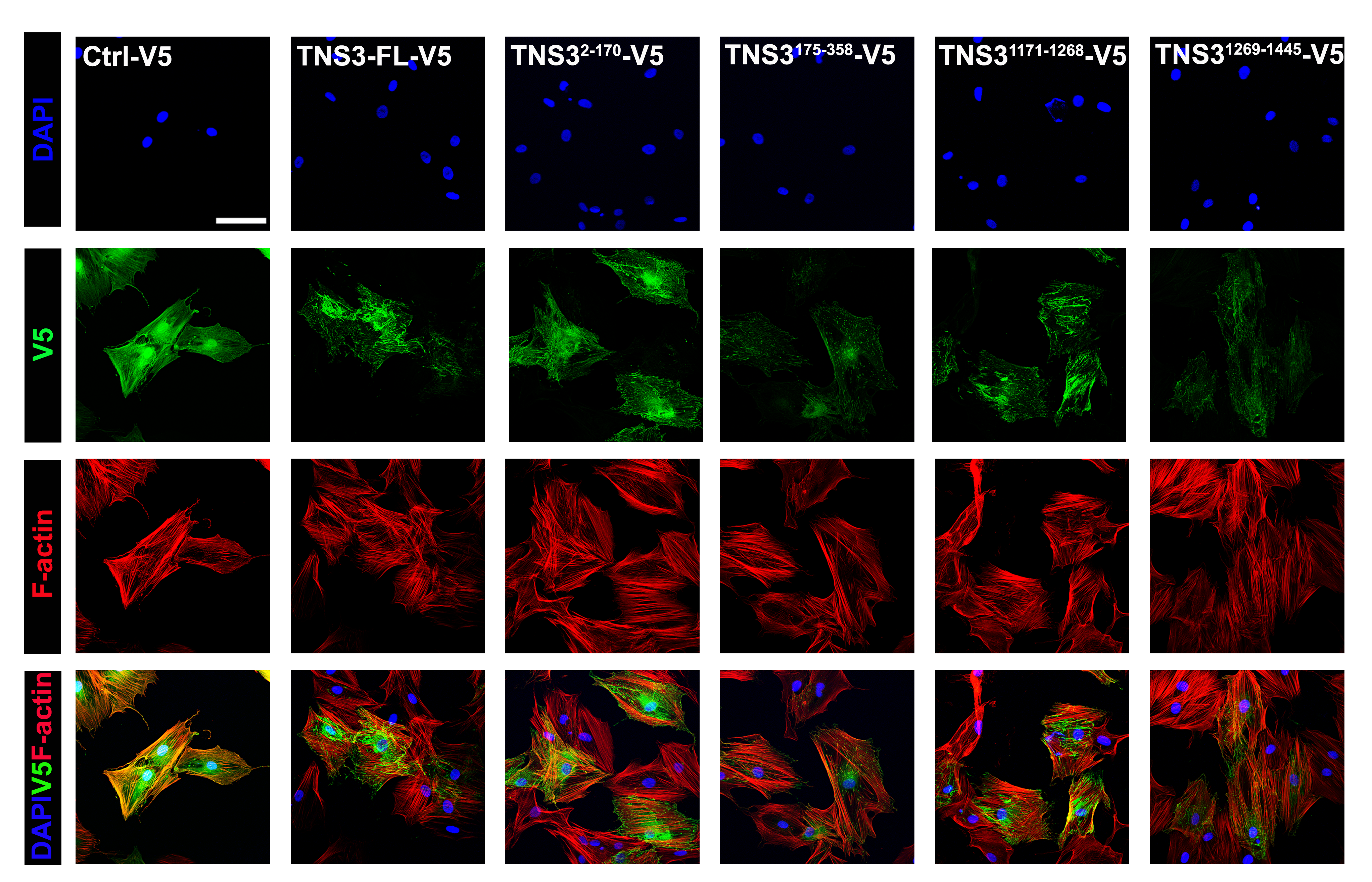

Supplement: Supplementary file 2 — Supplementary file2 (TIF 31486 KB) Fig.S2 Overexpression of TNS3 deletion mutants have similar intracellular localization as full-length TNS3. hMSCs expressing the indicated deletion constructs were immunostained with V5 (Alexa Fluor 488) and F-actin (phalloidin-rhodamine), and Nuclei (DAPI) after 3 days osteogenic induction. Scale bars: 200 μm [file 18_2023_4930_MOESM2_ESM.tif]

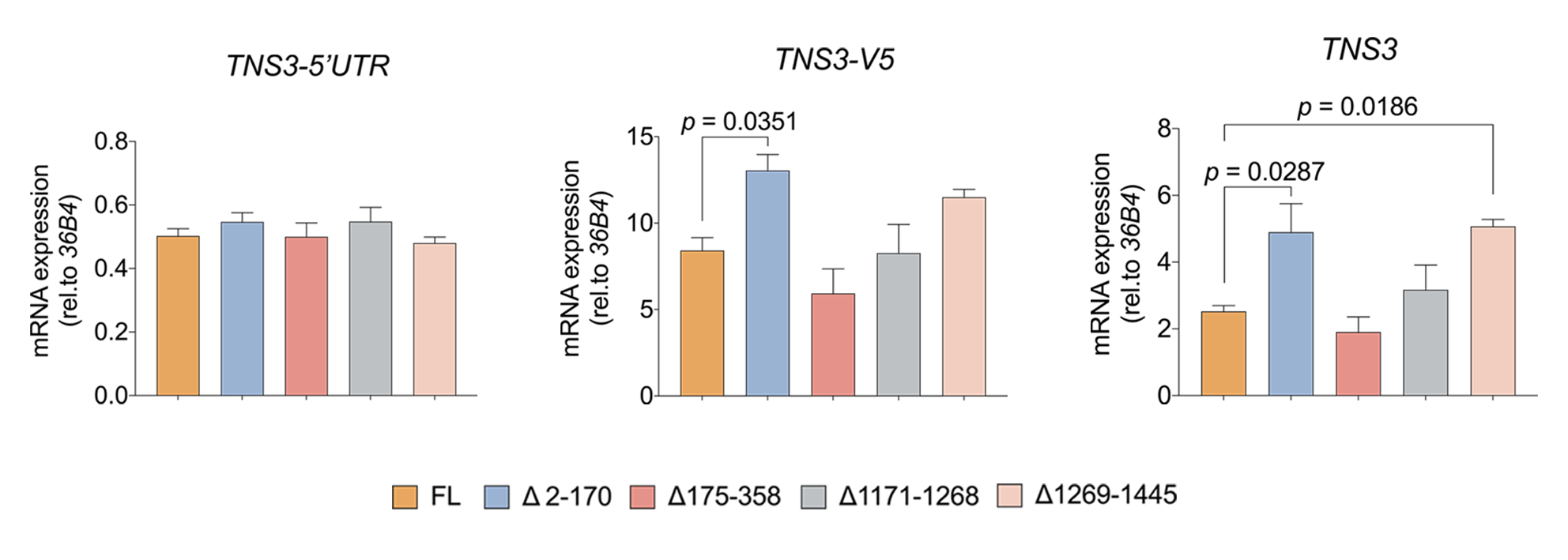

Supplement: Supplementary file 3 — Supplementary file3 (TIF 4142 KB) Fig.S3 The expression levels of TNS3 in deletion constructs. mRNA expression of TNS3 deletion constructs were assessed with qRT-PCR at day 3 using primers targeting TNS3-5’UTR (untranslated region), V5, TNS3. [file 18_2023_4930_MOESM3_ESM.tif]

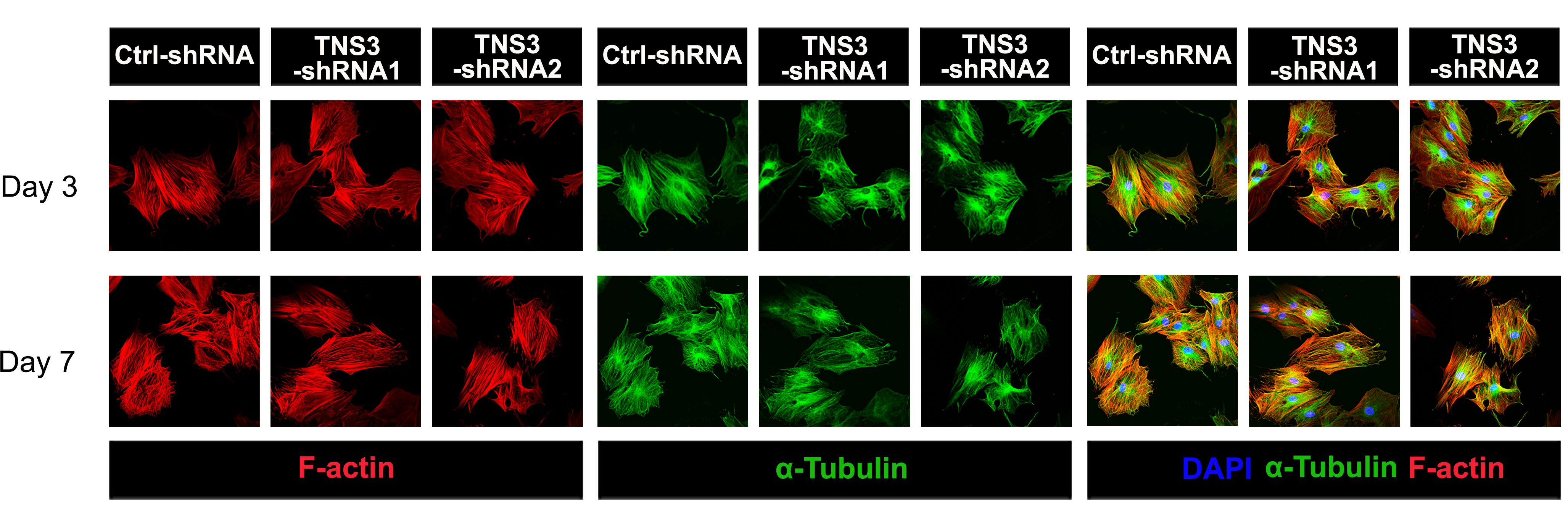

Supplement: Supplementary file 4 — Supplementary file4 (TIF 16519 KB) Fig.S4 TNS3 silencing in hMSCs affects cytoskeleton reorganization. A-C Confocal images of immunostainings against F-actin (phalloidin-rhodamine), α-tubulin (Alexa Fluor 488) and Nuclei (DAPI) after 3 days and 7 days following osteogenic induction. Scale bars: 200 μm [file 18_2023_4930_MOESM4_ESM.tif]

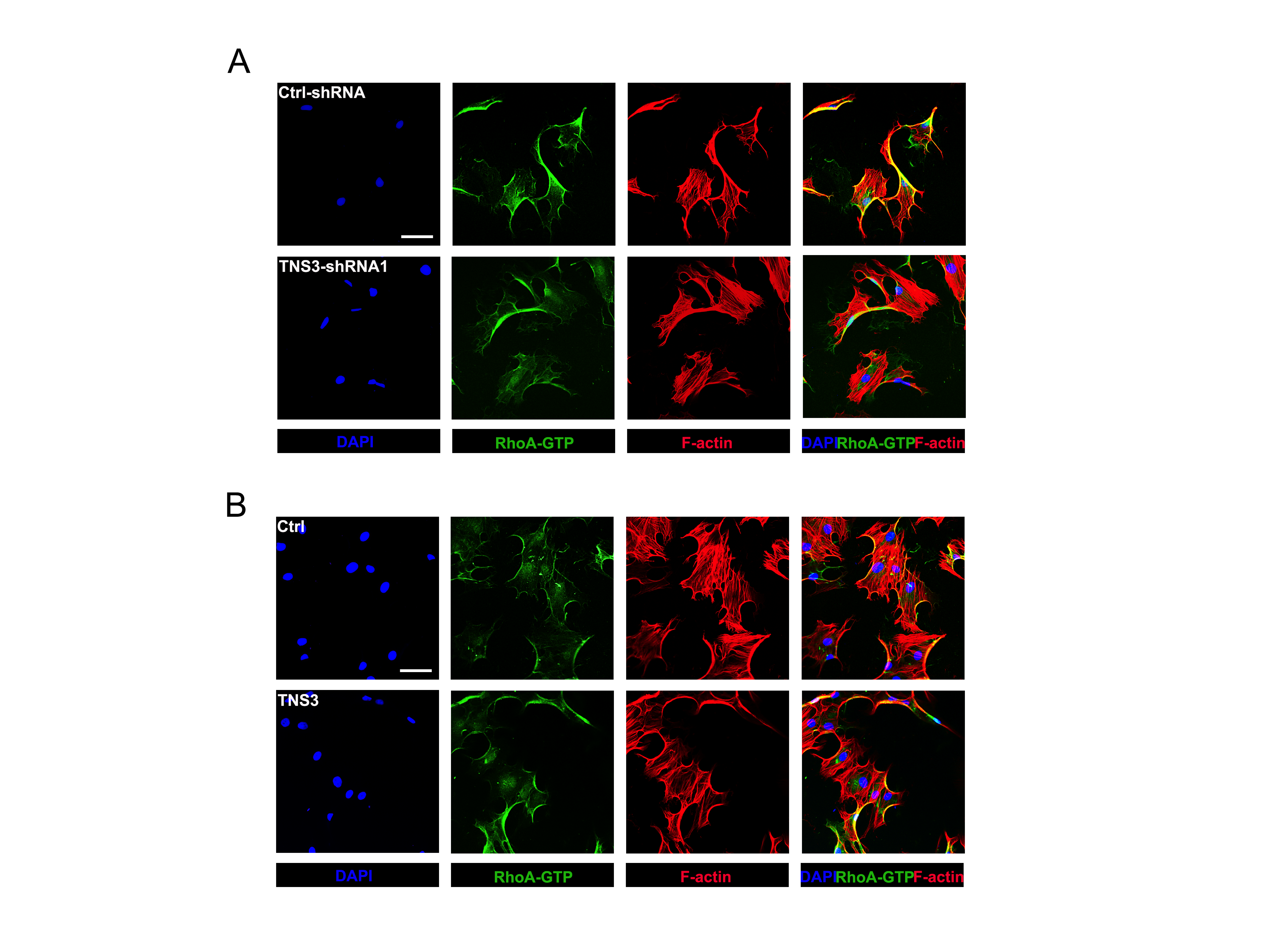

Supplement: Supplementary file 5 — Supplementary file5 (TIF 36148 KB) Fig.S5 TNS3 does not alter the co-localization of RhoA with the actin cytoskeleton. A-B Confocal images of immunostainings against F-actin (phalloidin-rhodamine), RhoA (Alexa Fluor 488) and Nuclei (DAPI) at 3 days after osteogenic induction following RNA silencing (A) or overexpression (B). Scale bars: 200 μm [file 18_2023_4930_MOESM5_ESM.tif]
