# Supplementary material for: Tensin-3 is involved in osteogenic versus adipogenic fate of human bone marrow stromal cells
Source: Cell Mol Life Sci. 2023 Sep 5;80(9):277. doi: 10.1007/s00018-023-04930-5 (PMC10480249; doi:10.1007/s00018-023-04930-5)
Supplement: Supplementary file 6 — Supplementary file6 (DOCX 33 KB) [file 18_2023_4930_MOESM6_ESM.docx]

Table S1. List of shRNAs used for TNS3

| Target gene | ID | Target sequence |
| --- | --- | --- |
| TNS3 | shRNA1 | CGTGTGGTACTTGAACTCTGT |
|  | shRNA2 | GTTCTGGTACAAGGCGGATAT |
| Nontargeting shRNA | ID | Oligonucleotide sequence |
| Ctrl | SHC002 | CCGGCAACAAGATGAAGAGCACCAACTCGAGTTGGTGCTCTTCATCTTGTTGTTTTT |
