# Supplementary material for: Tensin-3 is involved in osteogenic versus adipogenic fate of human bone marrow stromal cells
Source: Cell Mol Life Sci. 2023 Sep 5;80(9):277. doi: 10.1007/s00018-023-04930-5 (PMC10480249; doi:10.1007/s00018-023-04930-5)
Supplement: Supplementary file 7 — Supplementary file7 (DOCX 15 KB) [file 18_2023_4930_MOESM7_ESM.docx]

Table S3. Primer sequences used for q-RT PCR in this study

| Name | Sequence |
| --- | --- |
| *TNS3*-forward | ATAAGACGGAAGAGCGCCTG |
| *TNS3*-reverse | AGGATCTTCCAGGCCAAAGC |
| *BGLAP-* forward | CTCACACTCCTCGCCCTATTG |
| *BGLAP-* reverse | GCTTGGACACAAAGGCTGCAC |
| *SP7-* forward | CATCTGCCTGGCTCCTTG |
| *SP7-* reverse | GCCATAGTGAACTTCCTCCTCA |
| *SPP1-*forward | AGGCATCACCTGTGCCATAC |
| *SPP1-*reverse | CACAGCATTCTGCTTTTCCTCA |
| *PPARG*-forward | TCTCAAACGAGAGTCAGCCT |
| *PPARG*-reverse | CACGGAGCTGATCCCAAAGT |
| *FABP4*-forward | TACTGGGCCAGGAATTTGAC |
| *FABP4*-reverse | GGACACCCCCATCTAAGGTT |
| *PLIN1*-forward | CCTGCCTTACATGGCTTGTT |
| *PLIN1*-reverse | ATTCTCCTGCTCAGGGAGGT |
| *LPL*-forward | TTGGAGAAGCTATCCGCGTG |
| *LPL*-reverse | CGTGGGAGCACTTCACTAGC |
| *ADIPOQ*-forward | AACATGCCCATTCGCTTTACC |
| *ADIPOQ*-reverse | TTGGAGAAGCTATCCGCGTG |
| *ITGB1*-forward | TGGGCGCTGTCACGTC |
| *ITGB1*-reverse | TGTAAATTCATCTGCGCTTGCC |
| *CCN1*-forward | TGAAGCGGCTCCCTGTTTTT |
| *CCN1*-reverse | TGAGCACTGGGACCATGAAG |
| *TNS3*-5’UTR- forward | TCCAAAGTGTGTGGAGTTTGC |
| *TNS3*-5’UTR- reverse | AATACTTGCAGGCTCGGC |
| *36B4*-forward | GTCCTCGTGGAAGGCCC |
| *36B4*-reverse | AGGAGAGACAGGGAGCTCAG |
